# Integrative analysis of single nucleotide polymorphisms and gene expression efficiently distinguishes samples from closely related ethnic populations

**DOI:** 10.1186/1471-2164-13-346

**Published:** 2012-07-28

**Authors:** Hsin-Chou Yang, Pei-Li Wang, Chien-Wei Lin, Chien-Hsiun Chen, Chun-Houh Chen

**Affiliations:** 1 Institute of Statistical Science, Academia Sinica, 128, Academia Road, Section 2 Nankang, Taipei 115, Taiwan; 2 Institute of Biomedical Sciences, Academia Sinica, Taipei, Taiwan

**Keywords:** Single nucleotide polymorphism (SNP), Allele frequency, Gene expression, HapMap, Classification analysis, Ancestry informative marker (AIM)

## Abstract

**Background:**

Ancestry informative markers (AIMs) are a type of genetic marker that is informative for tracing the ancestral ethnicity of individuals. Application of AIMs has gained substantial attention in population genetics, forensic sciences, and medical genetics. Single nucleotide polymorphisms (SNPs), the materials of AIMs, are useful for classifying individuals from distinct continental origins but cannot discriminate individuals with subtle genetic differences from closely related ancestral lineages. Proof-of-principle studies have shown that gene expression (GE) also is a heritable human variation that exhibits differential intensity distributions among ethnic groups. GE supplies ethnic information supplemental to SNPs; this motivated us to integrate SNP and GE markers to construct AIM panels with a reduced number of required markers and provide high accuracy in ancestry inference. Few studies in the literature have considered GE in this aspect, and none have integrated SNP and GE markers to aid classification of samples from closely related ethnic populations.

**Results:**

We integrated a forward variable selection procedure into flexible discriminant analysis to identify key SNP and/or GE markers with the highest cross-validation prediction accuracy. By analyzing genome-wide SNP and/or GE markers in 210 independent samples from four ethnic groups in the HapMap II Project, we found that average testing accuracies for a majority of classification analyses were quite high, except for SNP-only analyses that were performed to discern study samples containing individuals from two close Asian populations. The average testing accuracies ranged from 0.53 to 0.79 for SNP-only analyses and increased to around 0.90 when GE markers were integrated together with SNP markers for the classification of samples from closely related Asian populations. Compared to GE-only analyses, integrative analyses of SNP and GE markers showed comparable testing accuracies and a reduced number of selected markers in AIM panels.

**Conclusions:**

Integrative analysis of SNP and GE markers provides high-accuracy and/or cost-effective classification results for assigning samples from closely related or distantly related ancestral lineages to their original ancestral populations. User-friendly BIASLESS (**B**iomarkers **I**dentification **a**nd **S**amp**les S**ubdivision) software was developed as an efficient tool for selecting key SNP and/or GE markers and then building models for sample subdivision. BIASLESS was programmed in R and R-GUI and is available online at http://www.stat.sinica.edu.tw/hsinchou/genetics/prediction/BIASLESS.htm.

## Background

Ancestry informative markers (AIMs) are the genetic markers carrying ancestral information for classifying samples from a specific population or various ethnic populations [1-12]. AIMs have been applied to various study areas, including population genetics, forensic sciences, medical genetics, and others. In population genetics, AIMs can be used to estimate the genetic diversity, population differentiation, and admixture proportions and thereby provide a more detailed understanding of the genetic background of study populations [2,4,5,7,13,14]. In forensic sciences, AIMs can be used to infer ancestral or continental origin and thereby assist with victim identification in a disaster situation or criminal identification in a venue [2,15,16]. In medical genetics, AIMs are useful for reducing false positives and false negatives in genetic association studies. On the one hand, AIMs can assist in adjusting for potential genetic substructures in a case–control association study and thereby reduce false positives (i.e., diminish spurious association) [3]. On the other hand, AIMs can also be used to construct homogeneous sample groups in a genetic association study and thereby reduce false negatives (i.e., diminish power loss) [2]. In addition, AIMs can provide complementary information for self-reported ethnicity. In contrast to self-reported ethnicity, which reflects an individual’s environment and culture, AIM-determined ethnicity inferred from genetic markers reflects genetic inheritance and make-up. In particular, self-reported ethnicity may be challenged when samples have been recruited from a geographic region in which the residents are highly admixed [9]. Therefore, AIM-determined ethnicity, rather than self-reported ethnicity, is recommended for genetic studies; admixture mapping using AIMs is especially suitable for highly admixed populations [17].

Short tandem repeat polymorphisms (STRPs) and single nucleotide polymorphisms (SNPs) are the most frequently used genetic markers for AIMs, and each has its own strengths [1,9,18]. Genotyping platforms for genome-wide STRP and SNP markers have been established but are not specific to AIMs, and this significantly increases the average genotyping cost for AIMs. This urgent need motivates the development of AIM panels that contain as much ancestral information as possible, while keeping the number of AIMs as low as possible. AIM panels with a small to moderate number of genetic markers have been constructed to discern samples from different populations, including Europeans [12], East Asians [11], African Americans [17], and European Americans [3,19], and different continents [2,4,5,9,16] at a more reasonable price.

Although a small to moderate number of SNPs or STRPs could provide promising discriminative power to distinguish a large ethnic discrepancy (e.g., subdivision of samples from Asia, Africa, and Europe), it becomes very challenging to classify samples from closely related ancestral lineage (e.g., two East-Asian populations such as Han Chinese and Japanese) using only a small number of SNPs. In an example of a previous classification study [20], the HapMap II Asian, African and European samples were separated with a classification accuracy of 0.97 based on 64 SNPs on average. The number of SNPs increased to 84 but the classification accuracy was reduced to 0.84 on average if Han Chinese and Japanese samples were further regarded as samples from different sub-Asian populations and classified with African and European samples jointly. This difficulty to classify the samples from proximate populations could be overcome by using a large number of genetic markers [3,21], while genotyping cost will increase significantly.

Gene expression (GE) microarray technology has advanced in the past 20 years. Previous studies have shown that GE also is polymorphic and heritable variation in humans [22,23]. Importantly, GE exhibits different genetic/genomic profiles in different ethnic populations [24]. Similar to SNP markers, GE markers may potentially provide ancestral information for discriminating samples from different ethnic populations. Of note, the different natures of SNP and GE markers may mean that GE provides information that is supplementary to SNP information: GE markers are quantitative attributes responsible for gene regulation, and SNP markers may act as semi-quantitative (e.g., a locus with an additive effect) or qualitative (e.g., a locus with a dominant or recessive effect) variables that can be attributed to DNA variation. Regarding the relationship between SNP and GE markers, the regulation of GE may be unrelated to DNA sequences, as with epigenetic mechanisms [25], or it may be associated with SNPs, as with expression quantitative trait locus (eQTL) [22,26-28]; nevertheless, even in the case of an eQTL, only a limited proportion of GE variation can be explained by the eQTL. Therefore, in this study, we proposed that integrative analysis of these two types of genetic markers (SNP and GE markers) may provide a more promising alternative for construction of a high-accuracy and cost-beneficial AIM panel than analysis of SNP or GE markers alone. To the best of our knowledge, few (if any) studies in the literature have integrated GE markers with SNP markers to aid in subdividing samples from different ethnic populations. In this study, we investigated the performance of SNP and GE markers in population genetics and evaluated the plausibility of sample classification using the combined resources of SNP and GE data.

## Methods

**Figure 1:**
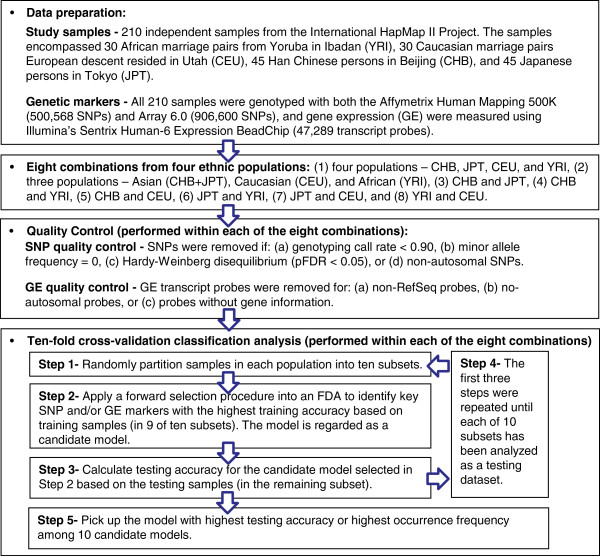
**Flowchart of this study.** A summary of data preparation, study combinations of ethnic populations, quality control of genetic markers, and classification analysis are shown.

A flowchart is provided to summarize the materials and analysis flow in this study (Figure 1).

### Samples and genotyping/gene expression experiments

In this study, we analyzed SNP and GE data in 210 independent samples from the International HapMap II Project [29-32]. The samples encompassed 30 African marriage pairs from Yoruba in Ibadan (YRI), 30 Caucasian marriage pairs of European descent resided in Utah (CEU), and 90 Asian persons including 45 Han Chinese persons in Beijing (CHB) and 45 Japanese persons in Tokyo (JPT). All 210 samples were genotyped using both the Affymetrix Human Mapping 500 K and Array 6.0 (Affymetrix Inc., Santa Clara, CA, USA). The two SNP gene chips provided genotype data for 500,568 SNPs and 906,600 SNPs, respectively, on 23 pairs of chromosomes for each individual. The Bayesian Robust Linear Model with Mahalanobis Distance Classifier (BRLMM) [33] or Birdseed [34] were used for SNP genotype call analysis of data from the Affymetrix Human Mapping 500 K and Array 6.0, respectively. The genotype data are publicly available (http://hapmap.ncbi.nlm.nih.gov/). In addition, GE levels of the 210 HapMap samples were measured using Illumina’s Sentrix Human-6 Expression BeadChip (Illumina Inc., San Diego, CA, USA). Each bead chip provided 47,289 transcript probes for the human genome [35,36]. Procedures for quantification and normalization of GE levels are described in Supporting Online Materials [35]. The normalized gene expression data are publicly available in the Gene Expression Omnibus (GEO) database (http://www.ncbi.nlm.nih.gov/geo/) (Series accession number GSE6536). Annotation of SNP data from the Affymetrix 500 K and Array 6.0 was derived from the NetAffx annotation update 30 (version: dbSNP Build 128), which is available on the Affymetrix website (http://www.affymetrix.com/). Annotation of GE probes was derived from the GEO annotation (accession number GPL2507; version: UCSC HG 18), which is available in the GEO database.

### Statistical methods and data analysis

This study classified samples in each of eight combinations of the four ethnic populations: (1) “four populations” – CHB, JPT, CEU, and YRI; (2) “three populations” – Asian (CHB + JPT), Caucasian (CEU), and African (YRI); (3) “CHB and JPT”; (4) “CHB and YRI”; (5) “CHB and CEU”; (6) “JPT and YRI”; (7) “JPT and CEU”; and (8) “YRI and CEU”. Quality control of SNP and GE markers was performed together with analysis for each of the eight combinations of ethnic populations. A poor-quality SNP was removed if its genotype call rate was lower than 0.9, its minor allele frequency was 0, or if Hardy-Weinberg equilibrium (HWE) was violated, where departure from HWE was defined as a p-value that was adjusted by a false discovery rate procedure [37] and that was lower than 0.05 in a permutation-based HWE test [38]. Finally, SNPs on sex chromosomes were removed. Quality control of GE markers removed 21,198 non-RefSeq probes (11,622 probes from UniGene and 9,576 probes from Gnomon). A total of 854 probes were removed from sex chromosomes, and 6,430 probes without gene information also were removed. The total numbers of SNP and GE markers that remained after SNP and GE quality control are shown ( Additional file 1: Table S1).

To explore the genetic discrepancy and sample subdivisions among the four HapMap II populations, an exploratory unsupervised analysis was performed, followed by an intensive supervised classification analysis. Both analyses used genome-wide SNP and GE markers. First, to understand whether genome-wide SNP and GE markers provide sufficient information for subdividing samples in HapMap II populations, a preliminary unsupervised classification analysis was performed by drawing allele frequency biplots and gene expression biplots based on genome-wide SNP and GE markers, respectively. The analysis was performed using ALOHA software [21], which is available on the ALOHA website (http://www.stat.sinica.edu.tw/hsinchou/genetics/aloha/ALOHA.htm). Afterward, intensive supervised classification analyses were performed to identify key SNP and/or GE markers to study subdivisions of samples from the HapMap II populations. A five-step discriminant analysis was developed to identify key SNP and/or GE markers with the highest prediction accuracy for the separation of samples from different populations as follows. First, samples in each study population were randomly partitioned into 10 subsets for cross-validation. Second, a flexible discriminant analysis (FDA) using optimal scoring [39] was applied to training sets (i.e., samples in nine of 10 subsets). Given the existing markers in a classification model, new SNP or GE markers with the maximum increment of training accuracy were added sequentially to the model. The marker with the minimum SSW/SSB was selected if more than one marker or marker set had the same training accuracy, where SSW and SSB indicate the within-population and between-population sum of squares for genotypic values or gene expression levels, respectively. The procedure continued until the training accuracy reached 1.0 or its increment was less than a threshold such as 0.001 in this study. Third, genetic markers with the highest training accuracy were used to classify individuals in the testing dataset (i.e., samples in the remaining subset) and the testing accuracy then was calculated. Fourth, the first three steps were repeated until each of the 10 subsets had been analyzed as a testing dataset, resulting in 10 classification candidate models. Finally, among the 10 classification models, the one with the highest testing accuracy or highest cross-validation consistency was selected as the best classification model. The aforementioned classification analysis was performed for each of the eight ethnic population combinations using only GE markers (“GE-only analysis”), only SNP markers on Affymetrix 500 K (“500 K-only analysis”), only SNP markers on Affymetrix Array 6.0 (“Array6.0-only analysis”), both GE markers and SNPs on Affymetrix 500 K (“500 K + GE analysis”), and both GE markers and SNPs on Affymetrix Array6.0 (“Array6.0 + GE analysis”). The analysis was performed using our developed software, BIASLESS (**B**iomarkers **I**dentification **a**nd **S**amp**les S**ubdivision), which can be downloaded for free at http://www.stat.sinica.edu.tw/hsinchou/genetics/prediction/BIASLESS.htm.

## Results

### Unsupervised classification analysis using genome-wide SNP or GE markers

**Figure 2:**
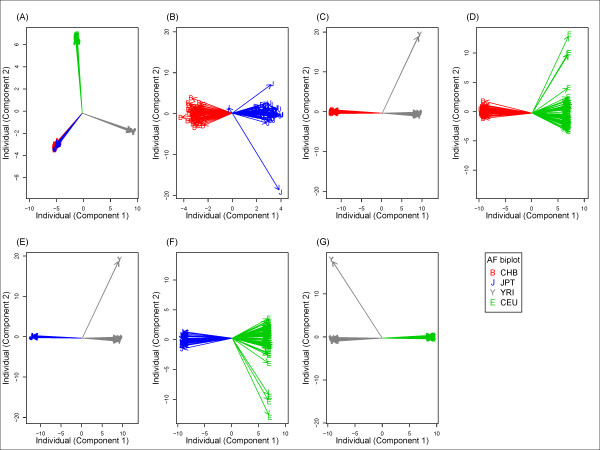
**Classification of HapMap samples using whole-genome SNPs of the Affymetrix Array 6.0 set.** All samples were superimposed onto a two-dimensional plane in an AF biplot. (**A**) CHB, JPT, CEU, and YRI, (**B**) CHB and JPT, (**C**) CHB and YRI, (**D**) CHB and CEU, (**E**) JPT and YRI, (**F**) JPT and CEU, and (**G**) YRI and CEU. Red line with a B symbol indicates CHB samples; blue line with a J symbol indicates JPT samples; gray line with a Y symbol indicates YRI samples; green line with an E symbol indicates CEU samples.

**Figure 3:**
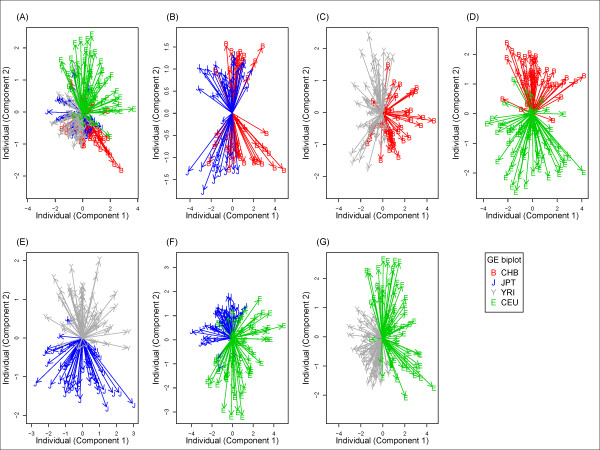
**Classification of HapMap samples using whole-genome gene expression.** All samples were superimposed onto a two-dimensional plane in a GE biplot. (**A**) CHB, JPT, CEU, and YRI, (**B**) CHB and JPT, (**C**) CHB and YRI, (**D**) CHB and CEU, (**E**) JPT and YRI, (**F**) JPT and CEU, and (**G**) YRI and CEU. Red line with a B symbol indicates CHB samples; blue line with a J symbol indicates JPT samples; gray line with a Y symbol indicates YRI samples; green line with an E symbol indicates CEU samples.

The genome-wide SNP-based classification analysis clearly separated samples from ethnic populations using allele frequency profiling of genome-wide SNPs interrogated on Affymetrix 500 K ( Additional file 2: Figure S1) or Affymetrix Array 6.0 (Figure 2). Samples from CHB, JPT, CEU, and YRI were classified into three genetically distant ethnic groups, African, Caucasian, and Asian. The Asian group consisted of two genetically close populations (CHB and JPT) ( Additional file 2: Figure S1A and Figure 2A) that were separated further by within-group analysis of Asian populations ( Additional file 2: Figure S1B and Figure 2B). All two-population analyses accurately separated samples from different populations ( Additional file 2: Figures S1B–G and Figure 2B–G). In general, the results of the Affymetrix 500 K and Affymetrix Array 6.0 analyses were very similar ( Additional file 2: Figure S1 and Figure 2). In contrast to the genome-wide SNP-based analysis, a non-negligible proportion of samples could not be separated correctly with genome-wide GE markers (Figure 3). These results were found not only for samples from the four populations (Figure 3A) but also for samples from any two populations (Figure 3B –G).

### Supervised classification analysis by selecting key predictive SNP and/or GE markers from genome-wide SNP and GE markers

**Figure 4:**
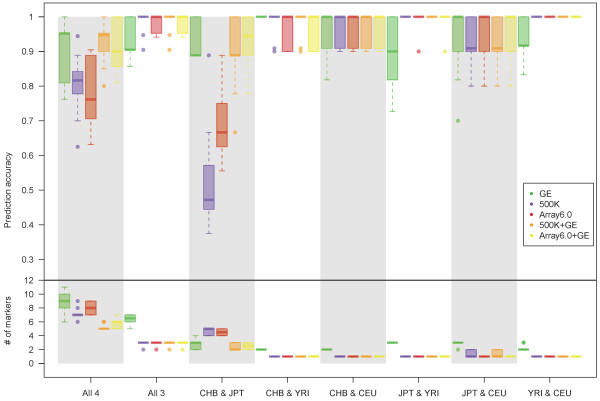
**Test accuracy and number of genetic markers in the selected classification models in the 10-fold cross-validation procedure.** In each of the eight ethnic population combinations, the testing accuracy and the number of genetic markers in the selected classification model in each of the 10-fold cross-validations are shown in the top and bottom panels, respectively. The top and bottom panels show box-whisker plots of the testing accuracy and the number of key predictive markers based on GE-only (green), 500 K-only (purple), Array6.0-only (red), 500 K + GE (orange), and Array6.0 + GE (yellow).

Ten classification models were established in each of the GE-only, 500 K-only, Array6.0-only, 500 K + GE, and Array6.0 + GE analyses, which independently identified a small number of key predictive SNP and/or GE markers to classify samples for each of the eight ethnic population combinations that we studied. The distributions of testing accuracy and number of predictive markers are presented in box-whisker plots (Figure 4). The majority of the classification analyses produced an average testing accuracy, calculated over 10 cross-validation datasets, greater than or close to 90%, with the exception of two SNP-only analyses; the 500 K-only and Array6.0-only analyses had relatively low testing accuracies for the classification of samples from two closely related ethnic populations, CHB and JPT. In the 500 K-only analysis, the average testing accuracies were only 0.53 and 0.70 for classifying samples from “CHB and JPT” and from “four populations”, respectively. Similarly, in the Array6.0-only analysis, the average testing accuracies were only 0.70 and 0.79 for the classification of samples from “CHB and JPT” and “four populations”, respectively. However, if GE markers also were integrated together with SNP markers for the classification of samples from “CHB and JPT” and “four populations”, the average testing accuracies increased to 0.89 and 0.92, respectively, in the 500 K + GE analysis and to 0.92 and 0.91 in the Array6.0 + GE analysis. In comparison with the integrative analyses of SNP and GE markers, the GE-only analysis presented a larger variation of testing accuracy and required about twice the number of markers to accurately classify samples from “JPT and YRI”, “YRI and CEU”, “three populations” and “four populations”.

We established the best classification models in the GE-only, 500 K-only, Array6.0-only, 500 K + GE, and Array6.0 + GE analyses for each of the eight ethnic population combinations we studied ( Additional file 3: Table S2). The best models of the integrative analysis of SNP and GE markers attained a testing accuracy of 100% in all eight population combinations that we studied. Only a few markers were needed for good sample classification. In the 500 K + GE analysis, the number of predictive markers was five for “four populations”, three for “three populations”, three for “CHB and JPT”, two for “JPT and CEU”, and one for the remaining population combinations; in the Array6.0 + GE analysis, the number of predictive markers required in the best model was five for “four populations”, three for “three populations”, three for “CHB and JPT”, and one for the remaining population combinations.

**Figure 5:**
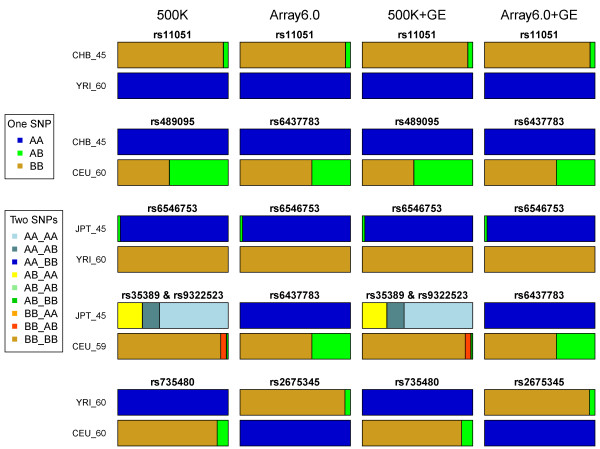
**Genotype frequencies of key predictive SNPs in the best model for the classification of samples from ethnically distant populations.** Bar charts show the genotype frequencies of the key predictive SNPs in the 500 K-only (first field), Array6.0-only (second field), 500 K + GE (third field), and Array6.0 + GE (fourth field) analyses for classification of samples from “CHB and YRI” (first panel), “CHB and CEU” (second panel), “JPT and YRI” (third panel), “JPT and CEU” (fourth panel), and “YRI and CEU” (fifth panel). Legends for the two scenarios, a single key predictive SNP or two key predictive SNPs, are shown. For the scenario of a single key predictive SNP, the proportion of blue, green, and brown reflects the genotype frequencies of AA, AB and BB of the SNP, respectively. The proportions range from 0 to 1 and sum to 1. For the scenario of two key predictive SNPs, nine colors are used to indicate nine possible genotype combinations for two SNPs. Again, the proportion of colored bars indicates the frequencies of genotype combinations, and the proportions range from 0 to 1 and sum to 1.

Notably, the best models in the 500 K + GE and Array6.0 + GE analyses only required 1 or 2 SNPs to correctly classify samples from genetically distant populations, including “CHB and YRI”, “CHB and CEU”, “JPT and YRI”, “JPT and CEU”, and “YRI and CEU”. The results show the existence of ancestry informative or population-specific SNPs; namely, the SNP-only analysis already provided key information, and GE markers were redundant in this situation, as follows: SNP rs11051 (G/A) for “CHB and YRI”, rs489095 (T/C) for “CHB and CEU”, rs6546753 (G/T) for “JPT and YRI”, rs6437783 (C/T) for “JPT and CEU”, and rs735480 (C/T) for “YRI and CEU” (Figure 5 and Additional file 4: Table S3). One or two SNPs already provided sufficient information for classifying ethnically distant samples, but this was not the case for classifying samples from ethnically close populations such as CHB and JPT. In the latter situation, the integrative analyses of SNP and GE markers indeed provided much richer information than the SNP-only analyses. The best model of the Affy500K + GE analysis, which was composed of GI_4506928-S (on *SH3GL1*), GI_37540521-S (on *OR13C5*), and rs11986045, significantly improved the testing accuracy of the Affy500K analysis when classifying samples from CHB and JPT; the testing accuracy of the best model increased from 0.89 to 1 ( Additional file 3: Table S2). The best model of the Array6.0 + GE analysis, which was composed of GI_4506928-S (on *SH3GL1*), GI_37540521-S (on *OR13C5*), and rs10485803, significantly improved the testing accuracy of the Array6.0 analysis; the testing accuracy of the best model increased from 0.89 to 1 ( Additional file 3: Table S2).

All samples from the four study populations could also be classified correctly with the best models of the integrative analyses of SNP and GE markers. In comparison with the SNP-only and GE-only analyses, the best integrative model of SNP and GE markers used only four SNP markers and one GE marker to perfectly classify samples from the four populations that we studied ( Additional file 3: Table S2). The 500 K + GE analysis prioritized rs2736306, GI_41281459-S (on *CENTB1*), rs12063564, rs2725379 (on *PURG*), and rs2250072 as the key predictive markers; the Array6.0 + GE analysis identified rs6546753, GI_4506928-S (on *SH3GL1*), rs1986420, rs6560625 and rs12632185 as the key predictive markers in the best classification model.

### BIASLESS software

The developed classification algorithm is packaged intoBIASLESS software with a user friendly interface programmed in language R and R-GUI (http://www.r-project.org/) ( Additional file 5: Figure S2). Programs, test examples, and the user guide are available at the BIASLESS website (http://www.stat.sinica.edu.tw/hsinchou/genetics/prediction/BIASLESS.htm). Before using BIASLESS software, users are encouraged to read the user guide for software installation, initialization, working directories, functions, operation, and format of input/output data. BIASLESS is structured by the following five main components:

(1) Input/Output settings: Users can choose between two types of data input formats (for markers). Users can click the browse buttons to specify the data input directory (for markers), data input directory (for a trait), and result output directory. All results will be saved automatically in the user-specified output directories. Users should fill in a notation or code to indicate any missing values in their marker data and trait data.

(2) Cross-validation: (a) Seed for cross-validations: users can use a random seed or provide a fixed seed (a real value between −65535 and 65535) for partitioning samples into the training dataset and testing dataset. (b) Fold size: users can select the number of cross-validations (e.g., “10” denotes a 10-fold cross-validation).

(3) Marker selection (Stop if any of the following three procedures stop): (a) Continue until the number of markers in the model increases to a specified value. (b) Continue until the training/testing accuracy increases to a specified value. (c) Continue until the increment of training/testing accuracy is reduced to a specified value.

(4) Graphical output: (a) Overlay line graph: the curves of training accuracy and testing accuracy are overlaid in a plot (the horizontal axis is the number of markers in the model and the vertical axis is the value of the accuracy) by cross-validation datasets. (b) Parallel coordinates plot: the number of markers selected, training accuracy, and testing accuracy over cross-validation datasets are presented in a parallel coordinates plot. (c) Multidimensional scaling plot: the study samples and selected markers in the final classification model with the highest testing accuracy are presented in a multidimensional scaling plot. (d) Stacked-bar/box-whisker plot: genotypic distributions (*AA* call – blue color, *AB* call – green color, and *BB* call – brown color) of SNPs and expression distributions (pink color) of genes selected in the classification model are displayed. (e) Sample misclassification plot: states of correct classification or misclassification in training and testing samples in each of 10-fold cross-validations are shown. Misclassification proportion across all cross-validations is shown for each individual in training and testing samples. Misclassification proportions across all training samples and all testing samples also are shown respectively for each step of marker selection in each of 10-fold cross-validations. (f) Marker impact plot: states of markers selected (red color) or unselected (blue color) in training samples in each of 10-fold cross-validations are shown. The figure also provides the selection times of the identified markers across all cross-validations and which step a marker is selected in a forward variable selection procedure. Graphical outputs in the test example of BIASLESS are provided ( Additional file 6: Figure S3).

## Discussion

The concept conveyed by the proposed integrative analysis of SNP and GE markers also is applicable to predicting disease status in biomedical studies and drug response in pharmacogenomics studies. Genome-wide association studies that identify disease susceptibility genes using a large number of SNPs suffer from the problem of missing heritability and are limited in explaining the etiology of complex diseases [40-42]. However, with the aid of GE, it is possible to increase the proportion of explained genetic variations which then elevates prediction accuracy. In view of the potential importance of integrative analysis of SNP and GE markers in the population genetics, forensic sciences, and medical genetics, we developed BIASLESS software. BIASLESS, which is useful for selecting important predictive marker sets from large numbers of biomarkers for inferences of ethnic groups, disease groups, and drug response groups, is a free, publicly available, and user-friendly analysis tool.

The method and software introduced in this paper can be used to construct high-accuracy and cost-beneficial AIM panels. Nevertheless, rather than the construction of AIM panels, the main focus of this paper is to introduce an integrative analysis of SNP and GE markers for the discrimination of samples from various populations, especially for closely related ancestral lineages. We don’t intend for the AIMs identified in this study to take the place of the AIMs found earlier for CEU, CHB, JPT, and YRI populations. Some of the AIMs identified in this study may be limited by the small to moderate number of samples in the HapMap II project; therefore, the generality of the identified AIMs should be further examined by using more independent samples and confirmed by biological verifications such as real-time reverse-transcription polymerase chain reaction before the AIMs applying to practical studies.

Although GE markers, which are more variable compared to SNPs, may change by population-specific food preferences or environmental exposures, previous studies did disclose the evidences of the genetic basis of global GE [28,36,43,44]. Moreover, this study analyzed the GE data from the total RNA samples extracted from Epstein Barr virus (EBV)-transformed lymphoblastoid cell lines of study individuals [35]. The GE variation of lymphoblastoid cell lines, which are important materials for dissecting genetic basis of GE variation of human populations [23,26,27,35,36,45], reflects a substantially higher proportion of genetic effect compared to the effect of food preferences or environmental exposures [46]. The finding of genetics of global GE can also be supported by previous studies. An important genomic study of global GE variation validated the genetic contribution of the discrepancy of GE between Asian and Caucasian samples, not an artifact due to life styles. This study showed that 24 Han Chinese residing in Los Angeles had much more similar GE profiles to the 82 HapMap CHB + JPT samples than to the 60 HapMap CEU samples [44]. The other important genomic study of GE also uncovered the genetic contribution on global patterns of GE after adjusting potential confounding factors that may influence GE. This study analyzed GE data of 270 individuals from four HapMap II populations and found GE variation differentiated in population comparisons in agreement with earlier studies [36].

The GE variation may also be influenced by the type of biological specimen, attributes related to the time and other circumstances of taking the biological samples, or GE microarray platform. This study provides a proof-of-concept method for construction of AIM panels by integrating SNP and GE markers but the current results are still limited by the use of single cell type (lymphoblastoid cell lines), fixed time/circumstances of taking the biological samples, and single microarray platform (Illumina’s Sentrix Human-6 Expression BeadChip). More investigations should be carried out to understand the proportions of the identified AIMs specific to the currently used conditions or transferable to more general conditions. For practical applications, we also plan to integrate SNP and GE variation from global genomic studies and construct larger reference database for normalizing GE data. SNP and GE markers will be integrated to identify AIMs and establish robust discriminant models using BIASLESS software. Biological specimen from a tested individual are collected and used to genotype/measure the identified and confirmed AIMs. Finally, SNP genotypes and GE levels of the tested individual are plugged into the discriminant models to determine the correct ethnic group.

Regarding the supervised classification method, two points are important to discuss. First, we modified the efficient and broadly used FDA algorithm and integrated forward variable selection and cross-validation procedures with FDA to select key predictive markers from enormous numbers of SNP and GE markers, and we then built accurate classification models for sample subdivision. Our supervised classification procedure provides multiple candidate models (e.g., 10 in a 10-fold cross-validation). Choosing a model with the highest testing accuracy is recommended but should not be the only criterion for model selection. Other optimal criteria and domain knowledge may need to be considered to determine the best model that satisfying both statistical properties and biological relevance. For example, the cross-validation consistency of a model among all candidate models may be used simultaneously, or genetic knowledge, biological relevance, and quality evaluation of genetic markers may also be integrated to assist in selection of the final classification model. Second, there is a very rich body of literature in the field of supervised classification, including support vector mechanisms [47] and classification trees [48]. Different algorithms have pros and cons in different study scenarios and data types. We are adding various classification algorithms to further enrich the BIASLESS software.

This study analyzed the data in the HapMap II Project, which contains only four populations, rather than the HapMap III Project, which contains 11 populations because GE data for the majority of samples in the HapMap III Project are not available. However, the proposed method and software can be applied in general to construct AIM panels for additional populations. The SNP data in this study came from two genotyping platforms: Affymetrix 500 K and Array6.0 SNP chips. The results of the sample classification were similar, although the number of SNPs interrogated on Affymetrix 500 K (~4 – 4.9 hundred thousand SNPs after quality control) was only about half the number in Array 6.0 (~7 – 8.7 hundred thousand SNPs after quality control), suggesting that the ancestral information in SNPs identified with Affymetrix Array6.0 is not more informative than that in SNPs identified with Affymetrix 500 K, with regard to the classification of samples in the HapMap II Project. Recently, whole-genome sequencing technology, in comparison with SNP microarrays, has become more common and has promoted the identification of new common SNPs and rare variants. Novel population-specific or ancestry-informative variants may be identified, and more eQTLs that contribute to genetic variation of ancestry informative GE may become available. It will be interesting to investigate if the bottleneck in a SNP-only analysis for discerning samples from closely related populations can be overcome using highly dense common SNPs and rare variants from massive parallel sequencing in the 1000 Genomes Project [49].

## Conclusion

In conclusion, we recommend SNP-only analysis for sample subdivision when the study samples come from ethnically distant populations such as Asian (CHB + JPT), African (YRI), and Caucasian (CEU); ancestry informative or population-specific SNPs provide sufficient information for sample classification in this situation, but population-specific SNPs may not be available or may be very hard to identify in ethnically close populations such as Chinese (CHB) and Japanese (JPT). Quantitative GE data, which are more variable than qualitative SNP data, are useful for sample classification after properly removing noisy GE markers. Note, however, that the GE-only analysis is still limited by slightly fluctuating testing accuracies and a larger number of predictive markers even when the samples are from ethnically distant populations. However, GE data do reveal important classification information supplemental to SNP data. Using an integration of SNP and GE markers, we established classification models with a reduced number of markers to accurately assign samples to the correct ethnic populations. Importantly, the genotyping cost is reduced because the number of required markers in an AIM panel is significantly diminished after inclusion of ancestry informative GE markers.

## Availability and requirements

The BIASLESS software, test examples, and user guide can be downloaded from the BIASLESS website: http://www.stat.sinica.edu.tw/hsinchou/genetics/prediction/BIASLESS.htm.

**Project name:** Biomarker identification and sample subdivision.

**Project home page:**http://www.stat.sinica.edu.tw/hsinchou/genetics/prediction/BIASLESS.htm.

**Operating system:** MS Windows®.

**Programming language:** Language R and R-GUI.

**Other requirements:** No.

**Any restrictions to use by non-academics:** On request and citation.

## Abbreviations

AF : Allele frequency; AIM : Ancestry informative marker; CEU : CEPH Utah residents; CHB : Han Chinese in Beijing; CVC : Cross-validation consistency; eQTL : Expression quantitative trait loci; FDA : Flexible discriminant analysis; GE : Gene expression; JPT : Japanese in Tokyo; BIASLESS : Biomarkers Identification and Samples Subdivision; SNP : Single nucleotide polymorphism; SSB : Sum of squares between populations; SSW : Sum of squares within population; STRP : Short tandem repeat polymorphism; YRI : Yoruba in Ibadan.

## Competing interests

The authors declare that they have no competing interests.

## Authors’ contributions

HCY conceived the study, developed the statistical methods, and prepared the manuscript and software user guide. PLW and CWL developed the BIASLESS software and analyzed the data with HCY. CHC and CHC contributed to the discussion and reviewed the manuscript. All authors have read and approved the final manuscript.

## Supplementary Material

Additional file 1 **Table S1.** The total numbers of SNP and GE markers remaining in the analysis after quality control. This table summarizes the number of SNP and GE markers in each analysis of the eight combinations of ethnic populations. After quality control, 18,807 GE markers, 403,067 – 486,092 SNPs in the Affymetrix Human Mapping 500 K set, and 700,682 – 868,434 SNPs in the Affymetrix Array 6.0 set remained. The number of SNPs in the intersection of the Affymetrix Human Mapping 500 K and Array 6.0 sets was 385,493 – 469,057. Click here for file

Additional file 2 **Figure S1.** Classification of HapMap samples using whole-genome SNPs of Affymetrix Human Mapping 500 K set. All samples were superimposed onto a two-dimensional plane in an allele frequency (AF) biplot. (A) CHB, JPT, CEU, and YRI, (B) CHB and JPT, (C) CHB and YRI, (D) CHB and CEU, (E) JPT and YRI, (F) JPT and CEU, and (G) YRI and CEU. Red line with a B symbol indicates CHB samples; blue line with a J symbol indicates JPT samples; gray line with a Y symbol indicates YRI samples; green line with an E symbol indicates CEU samples. Click here for file

Additional file 3 **Table S2.** The best classification models. This table summarizes the key predictive markers, testing accuracy, cross-validation consistency (CVC), and number of genetic markers in the best classification models in the analysis of each of the eight combinations of ethnic populations. Click here for file

Additional file 4 **Table S3.** Genotype frequencies of key predictive SNPs in the best model for the classification of samples from ethnically distant populations. This table summarizes genotype frequencies of the key predictive SNPs in the 500 K-only (first field), Array6.0-only (second field), 500 K + GE (third field) and Array6.0 + GE (fourth field) analyses for the classification of samples from “CHB and YRI” (first panel), “CHB and CEU” (second panel), “JPT and YRI” (third panel), “JPT and CEU” (fourth panel), and “YRI and CEU” (fifth panel). The genotype frequencies also are shown in Figure 5. Click here for file

Additional file 5 **Figure S2.** Interface of BIASLESS software. BIASLESS software programmed in R and R-GUI is a user-friendly tool for the identification of key predictive markers to classify samples from different populations/groups. Click here for file

Additional file 6 **Figure S3.** Graphical outputs in BIASLESS software. BIASLESS software outputs six graphs including (A) overlay line graph, (B) parallel coordinates plot, (C) multidimensional scaling plot, (D) stacked-bar/box-whisker plot, (E) sample misclassification plot, and (F) marker impact plot from the analysis of a test example (Detailed explanations to these graphs can be seen in the User Guide of BIASLESS, which can be downloaded at http://www.stat.sinica.edu.tw/hsinchou/genetics/prediction/BIASLESS.htm). Click here for file

## References

[B1] Rosenberg NA Li LM Ward R Pritchard JK Informativeness of genetic markers for inference of ancestry Am J Hum Genet 2003 73 6 1402 1422 10.1086/380416 14631557 PMC1180403

[B2] Halder I Shriver M Thomas M Fernandez JR Frudakis T A panel of ancestry informative markers for estimating individual biogeographical ancestry and admixture from four continents: utility and applications Hum Mutat 2008 29 5 648 658 10.1002/humu.20695 18286470

[B3] Seldin MF Price AL Application of ancestry informative markers to association studies in European Americans PLoS Genet 2008 4 1 e5 10.1371/journal.pgen.0040005 18208330 PMC2211545

[B4] Nassir R Kosoy R Tian C White PA Butler LM Silva G Kittles R Alarcon-Riquelme ME Gregersen PK Belmont JW An ancestry informative marker set for determining continental origin: validation and extension using human genome diversity panels BMC Genet 2009 10 39 19630973 10.1186/1471-2156-10-39 PMC2728728

[B5] Kosoy R Nassir R Tian C White PA Butler LM Silva G Kittles R Alarcon-Riquelme ME Gregersen PK Belmont JW Ancestry informative marker sets for determining continental origin and admixture proportions in common populations in America Hum Mutat 2009 30 1 69 78 10.1002/humu.20822 18683858 PMC3073397

[B6] Myles S Stoneking M Timpson N An assessment of the portability of ancestry informative markers between human populations BMC Med Genomics 2009 2 45 10.1186/1755-8794-2-45 19619313 PMC2719660

[B7] Paschou P Lewis J Javed A Drineas P Ancestry informative markers for fine-scale individual assignment to worldwide populations J Med Genet 2010 47 12 835 847 10.1136/jmg.2010.078212 20921023

[B8] Drineas P Lewis J Paschou P Inferring geographic coordinates of origin for Europeans using small panels of ancestry informative markers PLoS ONE 2010 5 8 e11892 10.1371/journal.pone.0011892 20805874 PMC2923600

[B9] Londin ER Keller MA Maista C Smith G Mamounas LA Zhang R Madore SJ Gwinn K Corriveau RA CoAIMs: a cost-effective panel of ancestry informative markers for determining continental origins PLoS ONE 2010 5 10 e13443 10.1371/journal.pone.0013443 20976178 PMC2955551

[B10] Kidd JR Friedlaender FR Speed WC Pakstis AJ De La Vega FM Kidd KK Analyses of a set of 128 ancestry informative single-nucleotide polymorphisms in a global set of 119 population samples Investig Genet 2011 2 1 1 10.1186/2041-2223-2-1 PMC3025953 21208434

[B11] Tian C Kosoy R Lee A Ransom M Belmont JW Gregersen PK Seldin MF Analysis of East Asia genetic substructure using genome-wide SNP arrays PLoS ONE 2008 3 12 e3862 10.1371/journal.pone.0003862 19057645 PMC2587696

[B12] Tian C Plenge RM Ransom M Lee A Villoslada P Selmi C Klareskog L Pulver AE Qi L Gregersen PK Analysis and application of European genetic substructure using 300 K SNP information PLoS Genet 2008 4 1 e4 10.1371/journal.pgen.0040004 18208329 PMC2211544

[B13] Serre D Paabo S Evidence for gradients of human genetic diversity within and among continents Genome Res 2004 14 9 1679 1685 10.1101/gr.2529604 15342553 PMC515312

[B14] Pritchard JK Stephens M Donnelly P Inference of population structure using multilocus genotype data Genetics 2000 155 2 945 959 10835412 10.1093/genetics/155.2.945 PMC1461096

[B15] Pomeroy R Duncan G Sunar-Reeder B Ortenberg E Ketchum M Wasiluk H Reeder D A low-cost, high-throughput, automated single nucleotide polymorphism assay for forensic human DNA applications AnBio 2009 395 1 61 67 10.1016/j.ab.2009.07.041 19646946

[B16] Phillips C Salas A Sanchez JJ Fondevila M Gomez-Tato A Alvarez-Dios J Calaza M Casares-de-Cal M Ballard D Lareu MV Inferring ancestral origin using a single multiplex assay of ancestry-informative marker SNPs Forensic Sci Int Genet 2007 1 3–4 273 280 19083773 10.1016/j.fsigen.2007.06.008

[B17] Tian C Hinds DA Shigeta R Kittles R Ballinger DG Seldin MF A genomewide single-nucleotide-polymorphism panel with high ancestry information for African American admixture mapping Am J Hum Genet 2006 79 4 640 649 10.1086/507954 16960800 PMC1592561

[B18] Kayser M de Knijff P Improving human forensics through advances in genetics, genomics and molecular biology Nat Rev Genet 2011 12 3 179 192 10.1038/nrg2952 21331090

[B19] Price AL Butler J Patterson N Capelli C Pascali VL Scarnicci F Ruiz-Linares A Groop L Saetta AA Korkolopoulou P Discerning the ancestry of European Americans in genetic association studies PLoS Genet 2008 4 1 e236 10.1371/journal.pgen.0030236 18208327 PMC2211542

[B20] Zhou N Wang L Effective selection of informative SNPs and classification on the HapMap genotype data BMC Bioinformatics 2007 8 484 10.1186/1471-2105-8-484 18093342 PMC2245981

[B21] Yang HC Lin HC Huang MC Li LH Pan WH Wu JY Chen YT A new analysis tool for individual-level allele frequency for genomic studies BMC Genomics 2010 11 1 415 10.1186/1471-2164-11-415 20602748 PMC2996943

[B22] Cheung VG Spielman RS The genetics of variation in gene expression Nat Genet 2002 32 Suppl 522 525 12454648 10.1038/ng1036

[B23] Cheung VG Conlin LK Weber TM Arcaro M Jen KY Morley M Spielman RS Natural variation in human gene expression assessed in lymphoblastoid cells Nat Genet 2003 33 3 422 425 10.1038/ng1094 12567189

[B24] Storey JD Madeoy J Strout JL Wurfel M Ronald J Akey JM Gene-expression variation within and among human populations Am J Hum Genet 2007 80 3 502 509 10.1086/512017 17273971 PMC1821107

[B25] Portela A Esteller M Epigenetic modifications and human disease Nat Biotechnol 2010 28 10 1057 1068 10.1038/nbt.1685 20944598

[B26] Morley M Molony CM Weber TM Devlin JL Ewens KG Spielman RS Cheung VG Genetic analysis of genome-wide variation in human gene expression Nature 2004 430 7001 743 747 10.1038/nature02797 15269782 PMC2966974

[B27] Stranger BE Forrest MS Clark AG Minichiello MJ Deutsch S Lyle R Hunt S Kahl B Antonarakis SE Tavare S Genome-wide associations of gene expression variation in humans PLoS Genet 2005 1 6 e78 10.1371/journal.pgen.0010078 16362079 PMC1315281

[B28] Rockman MV Kruglyak L Genetics of global gene expression Nat Rev Genet 2006 7 11 862 872 10.1038/nrg1964 17047685

[B29] The International HapMap Consortium The International HapMap Project Nature 2003 426 6968 789 796 10.1038/nature02168 14685227

[B30] The International HapMap Consortium Integrating ethics and science in the international HapMap project Nat Rev Genet 2004 5 6 467 475 10.1038/nrg1351 15153999 PMC2271136

[B31] The International HapMap Consortium A haplotype map of the human genome Nature 2005 437 7063 1299 1320 10.1038/nature04226 16255080 PMC1880871

[B32] The International HapMap Consortium A second generation human haplotype map of over 3.1 million SNPs Nature 2007 449 7164 851 861 10.1038/nature06258 17943122 PMC2689609

[B33] Affymetrix Inc BRLMM: An improved genotype calling method for the GeneChip human mapping 500K array set 2006

[B34] Korn JM Kuruvilla FG McCarroll SA Wysoker A Nemesh J Cawley S Hubbell E Veitch J Collins PJ Darvishi K Integrated genotype calling and association analysis of SNPs, common copy number polymorphisms and rare CNVs Nat Genet 2008 40 10 1253 1260 10.1038/ng.237 18776909 PMC2756534

[B35] Stranger BE Forrest MS Dunning M Ingle CE Beazley C Thorne N Redon R Bird CP de Grassi A Lee C Relative impact of nucleotide and copy number variation on gene expression phenotypes Science 2007 315 5813 848 853 10.1126/science.1136678 17289997 PMC2665772

[B36] Stranger BE Nica AC Forrest MS Dimas A Bird CP Beazley C Ingle CE Dunning M Flicek P Koller D Population genomics of human gene expression Nat Genet 2007 39 10 1217 1224 10.1038/ng2142 17873874 PMC2683249

[B37] Benjamini Y Hochberg Y Controlling the false discovery rate: A practical and powerful approach to multiple testing J R Statist Soc B 1995 57 1 289 300

[B38] Guo SW Thompson EA Performing the exact test of Hardy-Weinberg proportion for multiple alleles Biometrics 1992 48 2 361 372 10.2307/2532296 1637966

[B39] Hastie T Tibshirani R Buja A Flexible discriminant analysis by optimal scoring J Amer Statist Ass 1994 89 428 1255 1270 10.1080/01621459.1994.10476866

[B40] Maher B Personal genomes: The case of the missing heritability Nature 2008 456 7218 18 21 10.1038/456018a 18987709

[B41] Manolio TA Collins FS Cox NJ Goldstein DB Hindorff LA Hunter DJ McCarthy MI Ramos EM Cardon LR Chakravarti A Finding the missing heritability of complex diseases Nature 2009 461 7265 747 753 10.1038/nature08494 19812666 PMC2831613

[B42] Eichler EE Flint J Gibson G Kong A Leal SM Moore JH Nadeau JH Missing heritability and strategies for finding the underlying causes of complex disease Nat Rev Genet 2010 11 6 446 450 10.1038/nrg2809 20479774 PMC2942068

[B43] Emilsson V Thorleifsson G Zhang B Leonardson AS Zink F Zhu J Carlson S Helgason A Walters GB Gunnarsdottir S Genetics of gene expression and its effect on disease Nature 2008 452 7186 423 U422 10.1038/nature06758 18344981

[B44] Spielman RS Bastone LA Burdick JT Morley M Ewens WJ Cheung VG Common genetic variants account for differences in gene expression among ethnic groups Nat Genet 2007 39 2 226 231 10.1038/ng1955 17206142 PMC3005333

[B45] Cheung VG Spielman RS Ewens KG Weber TM Morley M Burdick JT Mapping determinants of human gene expression by regional and genome-wide association Nature 2005 437 7063 1365 1369 10.1038/nature04244 16251966 PMC3005311

[B46] Gibson G The environmental contribution to gene expression profiles Nat Rev Genet 2008 9 8 575 581 10.1038/nrg2383 18574472

[B47] Cortes C Vapnik V Support-vector networks MLear 1995 20 3 273 297

[B48] Breiman L Friedman JH Olshen RA Stone CJ Classification and Regression Trees 1984 Wadsworth & Brooks/Cole Advanced Books & Software, Belmont, CA

[B49] Siva N 1000 Genomes project Nat Biotechnol 2008 26 3 256 18327223 10.1038/nbt0308-256b

