# Supplementary material for: Integrative analysis of single nucleotide polymorphisms and gene expression efficiently distinguishes samples from closely related ethnic populations
Source: BMC Genomics. 2012 Jul 28;13:346. doi: 10.1186/1471-2164-13-346 (PMC3453505; doi:10.1186/1471-2164-13-346)
Supplement: Additional file 1 — Table S1. The total numbers of SNP and GE markers remaining in the analysis after quality control. This table summarizes the number of SNP and GE markers in each analysis of the eight combinations of ethnic populations. After quality control, 18,807 GE markers, 403,067 – 486,092 SNPs in the Affymetrix Human Mapping 500 K set, and 700,682 – 868,434 SNPs in the Affymetrix Array 6.0 set remained. The number of SNPs in the intersection of the Affymetrix Human Mapping 500 K and Array 6.0 sets was 385,493 – 469,057. [file 1471-2164-13-346-S1.doc]

**Table S1. The total numbers of SNP and GE markers remaining in the analysis after quality control.** This table summarizes the number of SNP and GE markers in each analysis of the eight combinations of ethnic populations. After quality control, 18,807 GE markers, 403,067 – 486,092 SNPs in the Affymetrix Human Mapping 500K set, and 700,682 – 868,434 SNPs in the Affymetrix Array 6.0 set remained. The number of SNPs in the intersection of the Affymetrix Human Mapping 500K and Array 6.0 sets was 385,493 – 469,057.

| **Population** | **GE** | **SNP** | | |
| --- | --- | --- | --- | --- |
|  |  | **500K** | **Array6.0** | **Intersection(500K, Array6.0)** |
| Four populations | 18,807 | 486,092 | 868,434 | 469,057 |
| CHB-JPT | 18,807 | 403,067 | 700,682 | 385,493 |
| CHB-YRI | 18,807 | 478,102 | 852,007 | 461,035 |
| CHB-CEU | 18,807 | 438,326 | 769,025 | 421,818 |
| JPT-YRI | 18,807 | 476,898 | 850,407 | 459,705 |
| JPT-CEU | 18,807 | 439,266 | 770,095 | 421,474 |
| YRI-CEU | 18,807 | 481,792 | 860,799 | 464,770 |
