# Supplementary material for: Integrative analysis of single nucleotide polymorphisms and gene expression efficiently distinguishes samples from closely related ethnic populations
Source: BMC Genomics. 2012 Jul 28;13:346. doi: 10.1186/1471-2164-13-346 (PMC3453505; doi:10.1186/1471-2164-13-346)

###### Figure S1. Classification of HapMap samples using whole-genome SNPs of Affymetrix Human Mapping 500K set. All samples were superimposed onto a two-dimensional plane in an allele frequency (AF) biplot. (A) CHB, JPT, CEU, and YRI, (B) CHB and JPT, (C) CHB and YRI, (D) CHB and CEU, (E) JPT and YRI, (F) JPT and CEU, and (G) YRI and CEU. Red line with a B symbol indicates CHB samples; blue line with a J symbol indicates JPT samples; gray line with a Y symbol indicates YRI samples; green line with an E symbol indicates CEU samples.


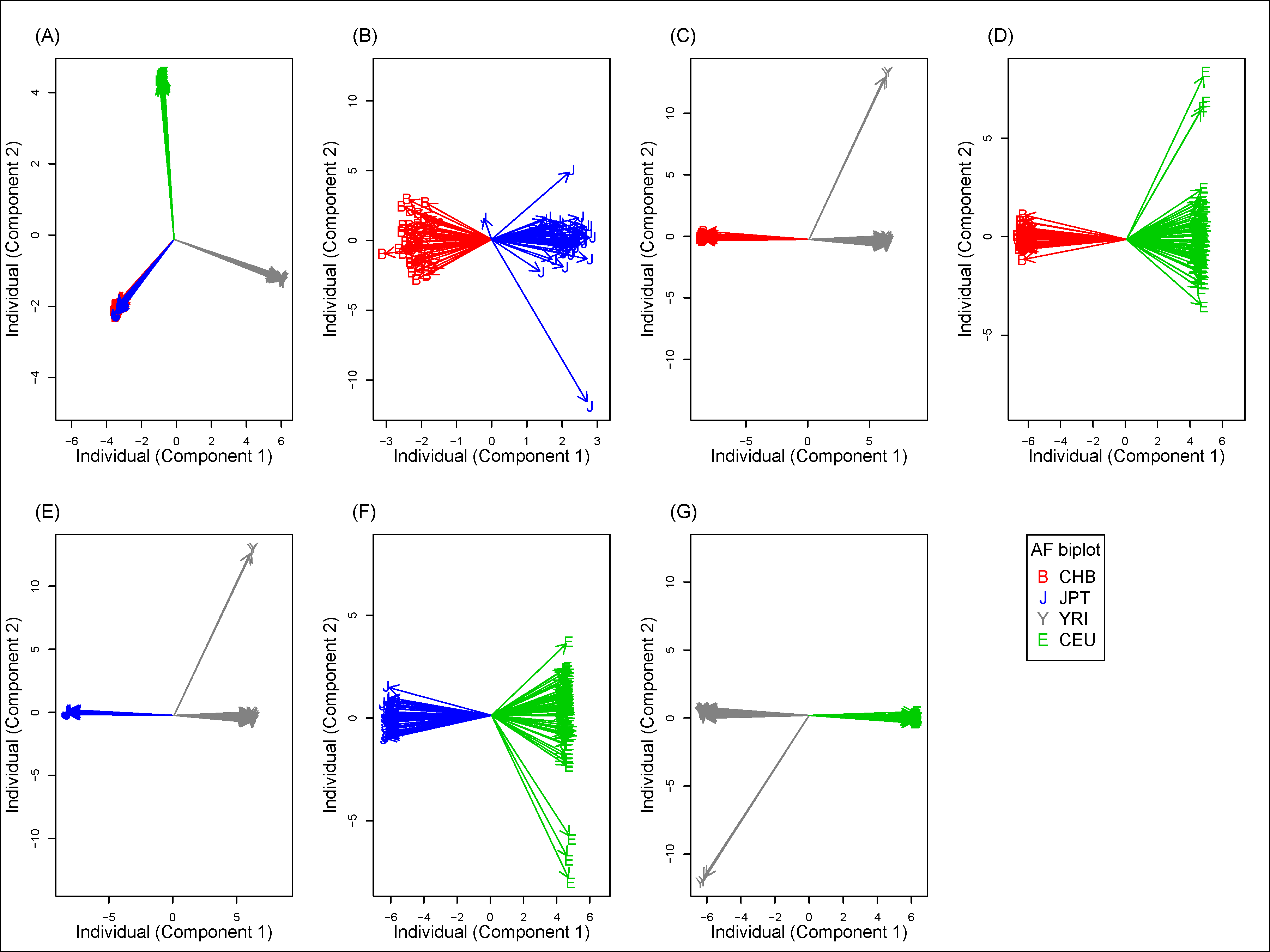

Supplement: Additional file 2 — Figure S1. Classification of HapMap samples using whole-genome SNPs of Affymetrix Human Mapping 500 K set. All samples were superimposed onto a two-dimensional plane in an allele frequency (AF) biplot. (A) CHB, JPT, CEU, and YRI, (B) CHB and JPT, (C) CHB and YRI, (D) CHB and CEU, (E) JPT and YRI, (F) JPT and CEU, and (G) YRI and CEU. Red line with a B symbol indicates CHB samples; blue line with a J symbol indicates JPT samples; gray line with a Y symbol indicates YRI samples; green line with an E symbol indicates CEU samples. [file 1471-2164-13-346-S2.doc]
