# Supplementary material for: Integrative analysis of single nucleotide polymorphisms and gene expression efficiently distinguishes samples from closely related ethnic populations
Source: BMC Genomics. 2012 Jul 28;13:346. doi: 10.1186/1471-2164-13-346 (PMC3453505; doi:10.1186/1471-2164-13-346)

###### Figure S2. Interface of BIASLESS software. BIASLESS software programmed in R and R-GUI is a user-friendly tool for the identification of key predictive markers to classify samples from different populations/groups.


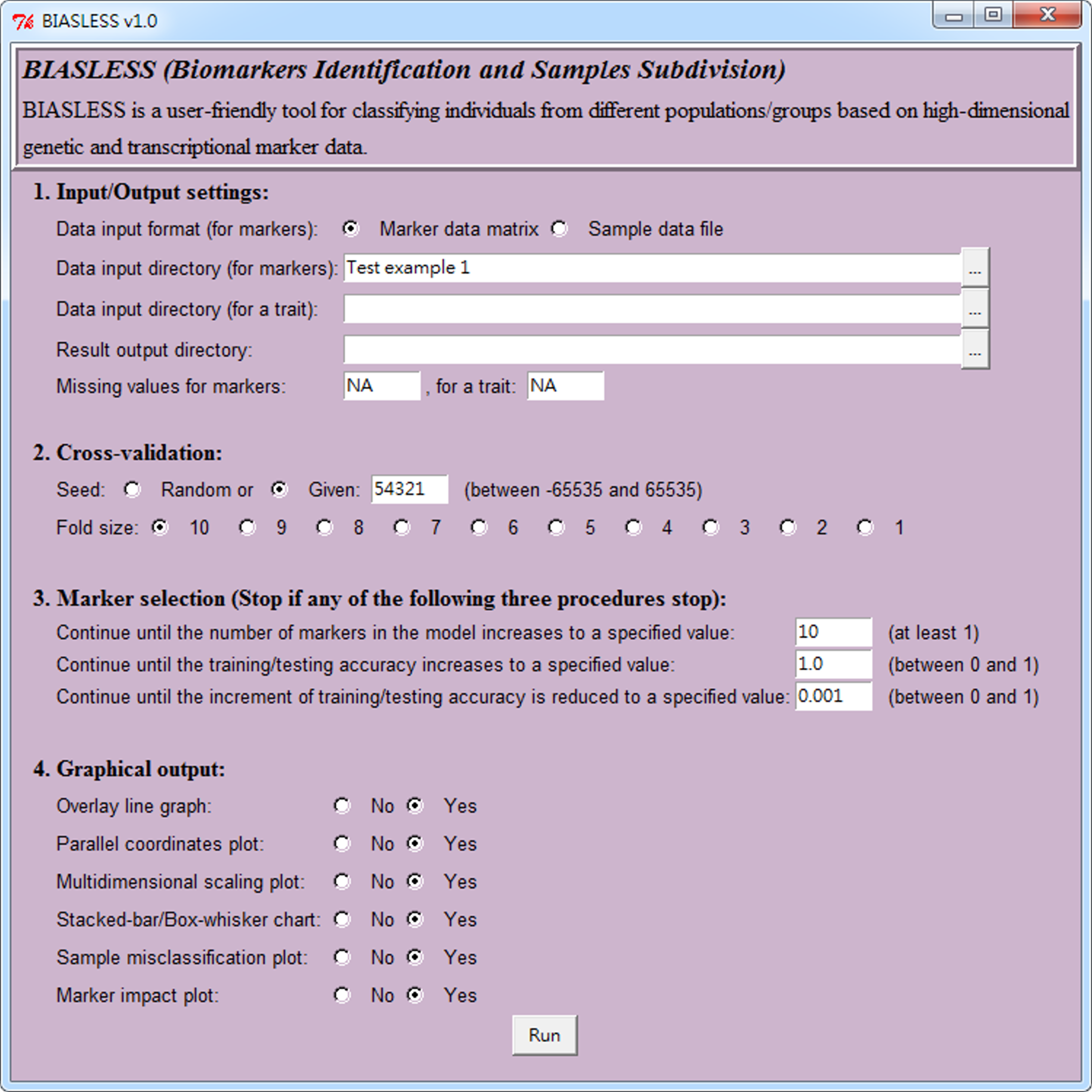

Supplement: Additional file 5 — Figure S2. Interface of BIASLESS software. BIASLESS software programmed in R and R-GUI is a user-friendly tool for the identification of key predictive markers to classify samples from different populations/groups. [file 1471-2164-13-346-S5.doc]
