# Supplementary material for: Integrative analysis of single nucleotide polymorphisms and gene expression efficiently distinguishes samples from closely related ethnic populations
Source: BMC Genomics. 2012 Jul 28;13:346. doi: 10.1186/1471-2164-13-346 (PMC3453505; doi:10.1186/1471-2164-13-346)

**Fig S3.** **Graphical outputs in BIASLESS software.** BIASLESS software outputs six graphs including (A) overlay line graph, (B) parallel coordinates plot, (C) multidimensional scaling plot, (D) stacked-bar/box-whisker plot, (E) sample misclassification plot, and (F) marker impact plot from the analysis of a test example (Detailed explanations to these graphs can be seen in the User Guide of BIASLESS, which can be downloaded at http://www.stat.sinica.edu.tw/hsinchou/genetics/prediction/BIASLESS.htm).

(A)


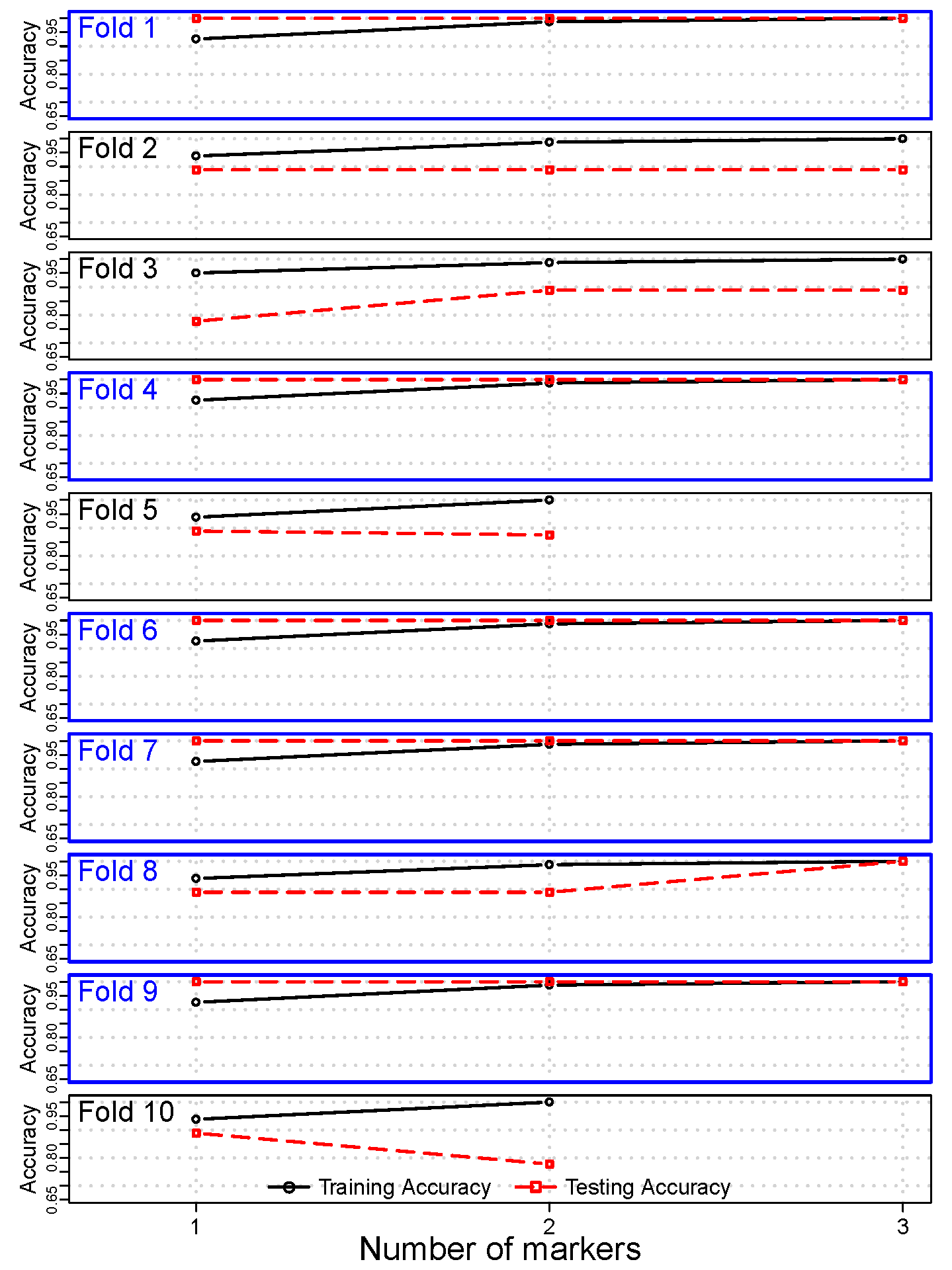


(B)


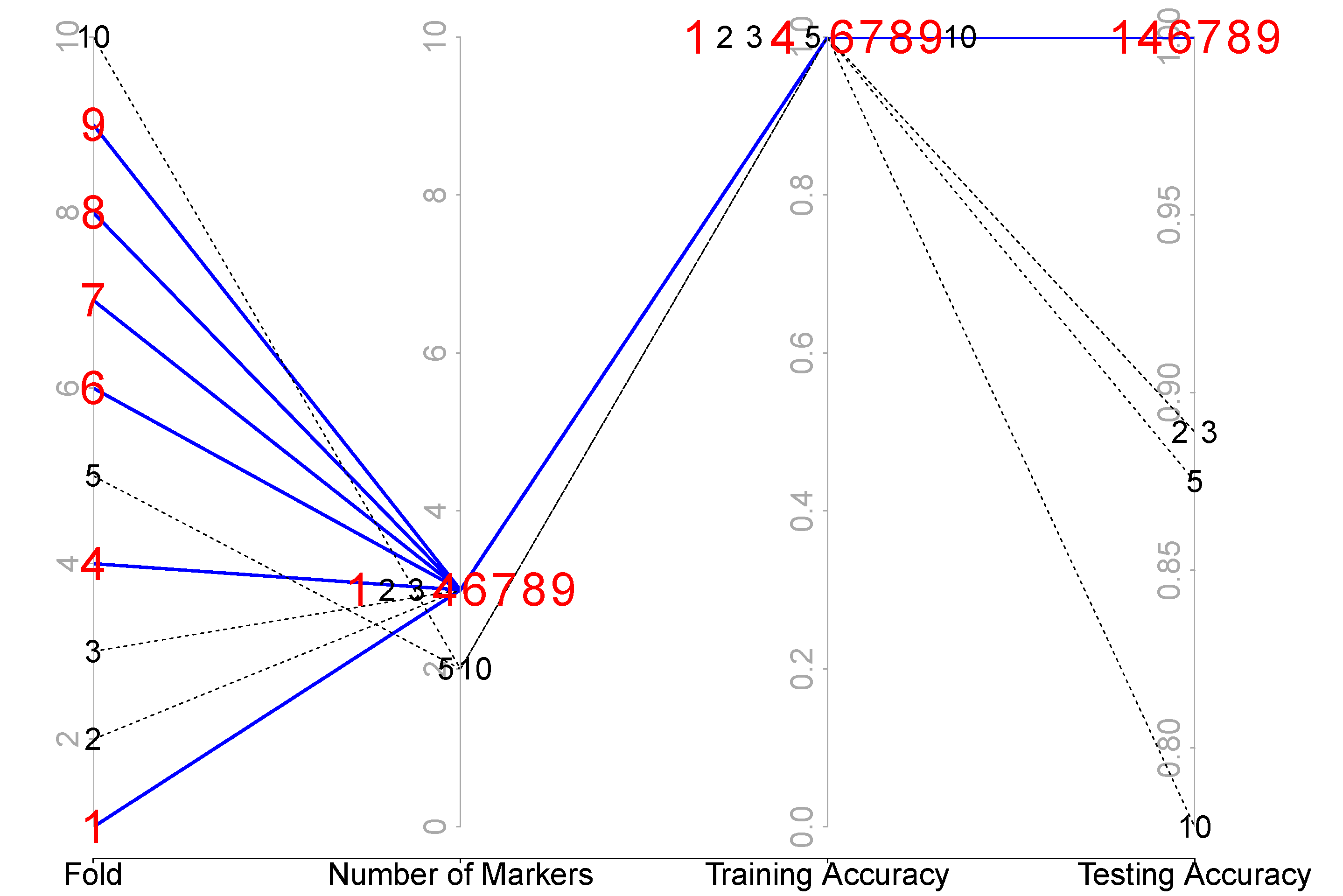


(C)

**
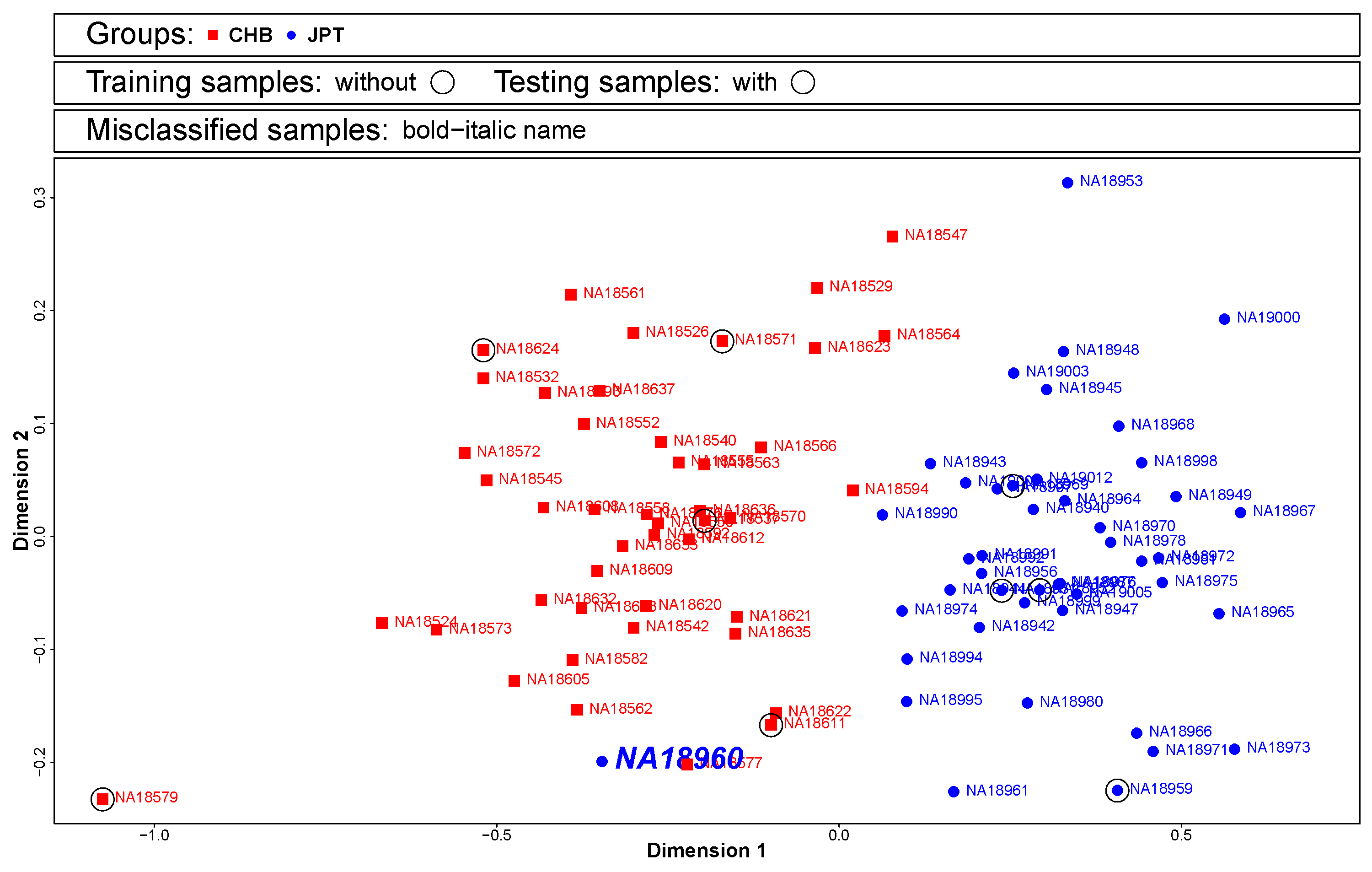
**

(D)


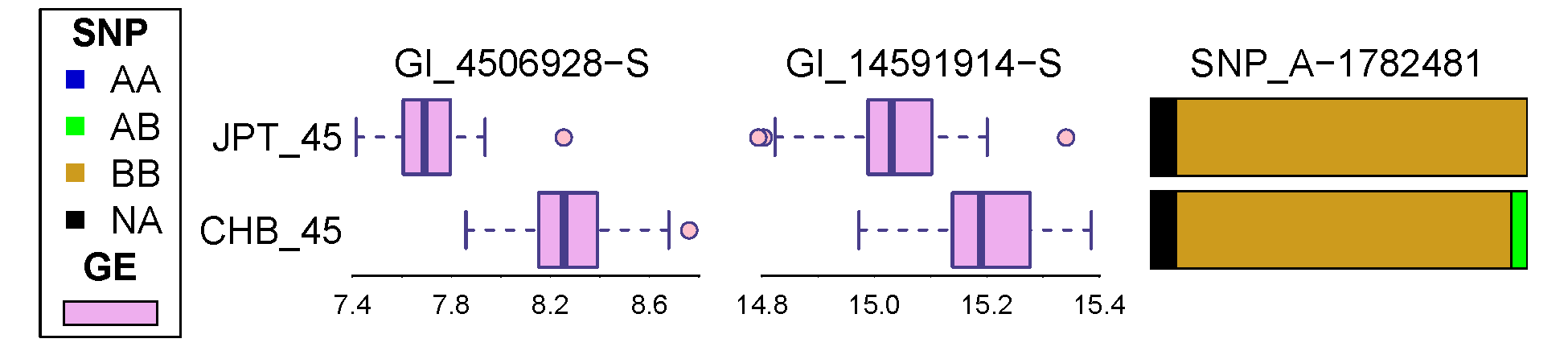


(E)


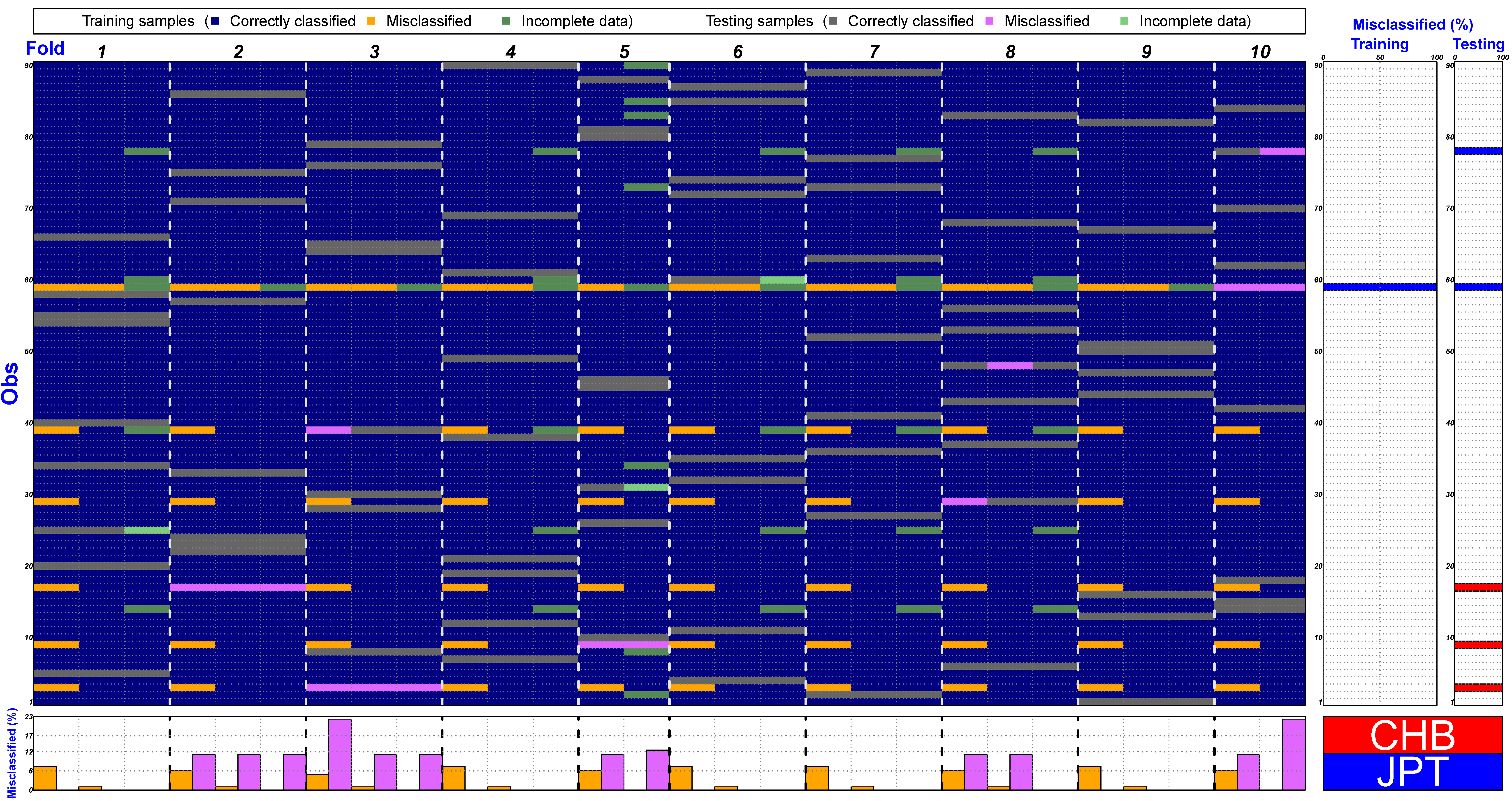


(F)


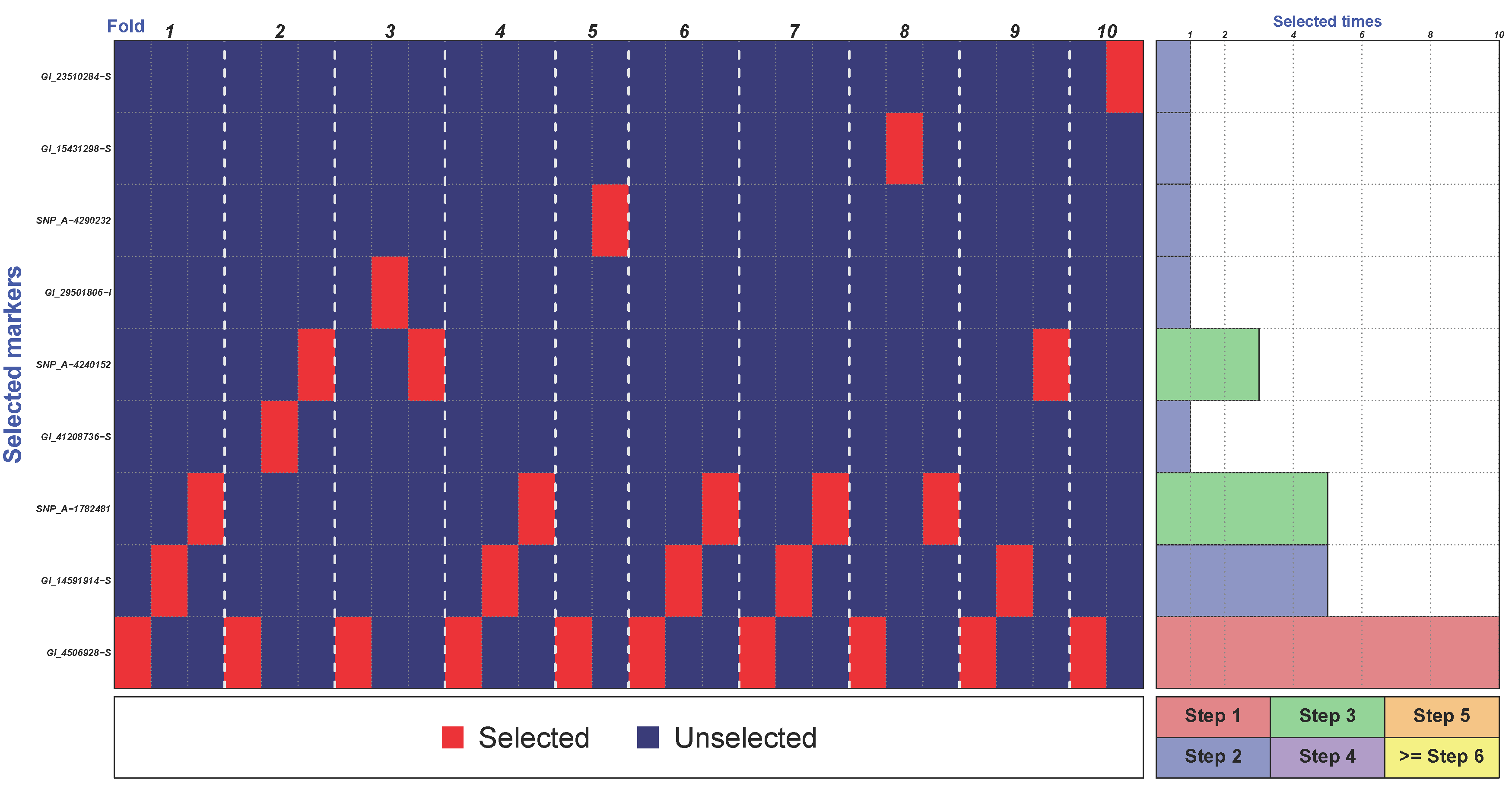

Supplement: Additional file 6 — Figure S3. Graphical outputs in BIASLESS software. BIASLESS software outputs six graphs including (A) overlay line graph, (B) parallel coordinates plot, (C) multidimensional scaling plot, (D) stacked-bar/box-whisker plot, (E) sample misclassification plot, and (F) marker impact plot from the analysis of a test example (Detailed explanations to these graphs can be seen in the User Guide of BIASLESS, which can be downloaded at http://www.stat.sinica.edu.tw/hsinchou/genetics/prediction/BIASLESS.htm). [file 1471-2164-13-346-S6.doc]
